# Utility of Quantitative and Semi-Quantitative SPECT/CT Metrics in Differentiating Mueller–Weiss Syndrome

**DOI:** 10.3390/diagnostics16010018

**Published:** 2025-12-20

**Authors:** Yi-Ching Lin, Shih-Chuan Tsai, Chia-Hung Kao, Shun-Ping Wang

**Affiliations:** 1 Department of Nuclear Medicine, Taichung Veterans General Hospital, Taichung 407, Taiwan; dianayjlin@gmail.com (Y.-C.L.); sctsai@vghtc.gov.tw (S.-C.T.); 2 Department of Medical Imaging and Radiological Sciences, Central Taiwan University of Science and Technology, Taichung 406, Taiwan; 3 Department of Post-Baccalaureate Medicine, College of Medicine, National Chung Hsing University, Taichung 402, Taiwan; 4 Department of Nuclear Medicine, China Medical University, Taichung 404, Taiwan; dr.kaochiahung@gmail.com; 5 Artificial Intelligence Center, China Medical University Hospital, China Medical University, Taichung 404, Taiwan; 6 Graduate Institute of Biomedical Sciences, School of Medicine, College of Medicine, China Medical University, Taichung 404, Taiwan; 7 Department of Bioinformatics and Medical Engineering, Asia University, Taichung 413, Taiwan; 8 Department of Orthopaedics, Taichung Veterans General Hospital, Taichung 407, Taiwan

**Keywords:** Mueller–Weiss, navicular, Maceira classification, SUV, bone scan, SPECT/CT, quantitative, semi-quantitative

## Abstract

**Background/Objectives:** Mueller–Weiss syndrome (MWS) is a rare condition characterized by spontaneous adult-onset osteonecrosis of the navicular bone. This study aimed to assess the diagnostic value of quantitative and semi-quantitative standardized uptake value (SUV) measurements on Tc-99m MDP SPECT/CT for differentiating MWS from other foot pathologies. **Methods:** We retrospectively reviewed 21 MWS patients who underwent SPECT/CT and compared them with 10 feet from 5 non-MWS patients as controls. MWS severity was staged using the Maceira classification. Volumes of interest (VOIs) were defined in the lateral navicular and distal tibia. SUV_max_ values were measured for the navicular bone (N), tibial metaphysis (Tm), and diaphysis (Td). Uptake ratios (N/Tm and N/Td) were calculated for semi-quantitative comparison. **Results:** MWS patients showed significantly higher SUV_max_ in the navicular compared with controls (9.2 vs. 1.5, *p* < 0.001). Both N/Tm and N/Td ratios were also significantly elevated (*p* < 0.001). SUV_max_ and uptake ratios positively correlated with Maceira stage and visual navicular uptake intensity. Diagnostic thresholds of N SUV_max_ > 3.77 (AUC = 0.93), N/Tm > 1.139 (AUC = 0.95), and N/Td > 0.93 (AUC = 0.93) effectively distinguished MWS from non-MWS cases. **Conclusions:** Quantitative and semi-quantitative SUV analysis on SPECT/CT offers a reliable tool for diagnosing MWS and evaluating disease severity. Semi-quantitative ratios, by normalizing metabolic variability, provide a practical and reproducible alternative to absolute SUV measurements for early detection and treatment planning in MWS.

## 1. Introduction

Mueller–Weiss Syndrome (MWS) is a rare foot condition characterized by spontaneous osteonecrosis or anomalous ossification of the lateral part of tarsal navicular in adults [1]. With the disease progression, the navicular deformity, along with bone fragmentation and the following structural collapse of the bone architecture of feet, will be identified on radiographic images and classified as five stages by Maceira et al. in 2004 [2]. However, while MWS often affects both feet, recent studies have unveiled variations in disease progression and the non-simultaneous onset of symptoms [3]. The subtle or no changes in the early stages of MWS on X-ray or CT scan can be challenging to be detected, potentially leading to delayed or missed diagnoses [4]. Moreover, the chronic, insidious foot pain in MWS can mimic symptoms caused by other foot pathologies, including Charcot foot, stress fractures, or osteomyelitis [5]. The bone scintigraphy with high sensitivity for early detection of MWS will be indispensable.

Bone scintigraphy with Tc-99m/labeled methylene diphosphonate (Tc-99m MDP), using conventional planar images, has long served as a well-established diagnostic tool. It provides high sensitivity for detecting lesions with abnormal bone turnover, primarily reflecting osteogenic activity [6,7,8,9]. However, the primary approach for interpreting conventional bone scintigraphy relies on visual assessment of hot spots on planar images. The lack of quantification of abnormal isotope uptake has resulted in noteworthy inter-reader discrepancies, thereby constraining its clinical utility [10,11]. The abnormal uptake of bone scintigraphy was correlated with the progression of skeletal disorders, recurrence, or metastasis, and quantification has been attempted since long time ago [12,13]. The complexity of quantifying radiotracer uptake on interesting targets that require the aid of in-house software and deep learning algorithms still impede its widespread and used in-routine clinical practice [13,14,15].

In comparison to isolated Tc-99m MDP bone scintigraphy, employing through single photon emission computed tomography/computed tomography (SPECT/CT) acquisition introduces a hybrid imaging modality, revealing a blend of metabolic and structural information. Tc-99m MDP with SPECT/CT enhanced their sensitivity and specificity for evaluating many various skeletal disorders included in bone tumors, metastatic malignancy, fractures, arthritis, and infectious processes [16,17]. The measurement of standardized uptake value (SUV) of radiotracer on bone scintigraphy, in combination with SPECT/CT, offers significant advantages for enhancing lesion localization. It also provides the capability to quantify abnormal bone metabolism [18,19]. However, to date, there has still been the paucity of research on the utilization of Tc-99m MDP SPECT/CT for detecting and quantifying the lesion of MWS. Furthermore, given the rarity of MWS cases, the applications of preoperative SPECT/CT, whether in conjunction with conventional planar imaging or as a standalone approach, still remain controversial. We initially introduced the semi-quantitative ratio of SPECT/CT, an alternative easier than direct quantification of SUV, to correlate with the staging of MWS cases and their radiographic findings.

This study aimed to evaluate the feasibility to utilize the quantitative and semi-quantitative ratio of SUV uptake on Tc-99m MDP SPECT/CT images to be a diagnostic aid for MWS. The research findings will offer clinicians a guide for making decisions regarding further treatment and the prediction of functional outcomes. We hypothesize that the quantified values yielded from SPECT/CT images can effectively identify the MWS lesion.

## 2. Materials and Methods

### 2.1. Patients’ Enrollment

We conducted a retrospective review of all consecutive adult patients aged over 18 radiographically diagnosed as MWS patients, who had undergone Tc-99m MDP SPECT/CT scans at our institution between November 2020 and November 2021. All MWS cases were confirmed by experienced foot and ankle surgeon based on the lateral and dorsoplantar (DP) foot standing radiographs, CT scans, or magnetic resonance images. The feet of MWS patients with a prior history of surgical intervention with implants for MWS were excluded from the study. In total, 21 patients with radiographically confirmed MWS were enrolled in this investigation.

**Figure 1:**
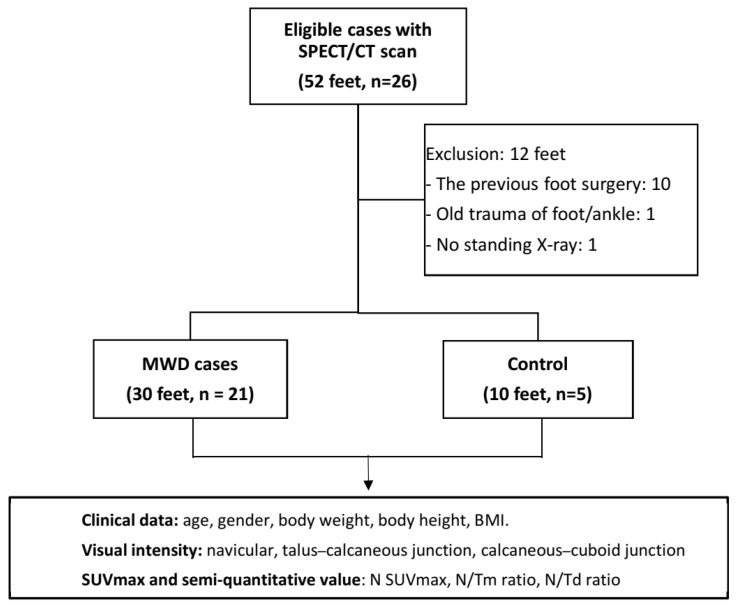
Flowchart of enrolled cases.

To establish a control group, we also included 10 feet in five patients who had undergone Tc-99m MDP SPECT/CT scans of their feet due to other pathological conditions, such as enthesopathy of the Achilles tendon or sesamoiditis. All control subjects exhibited no midfoot symptoms or imaging abnormalities involving the navicular or adjacent midfoot joints, and their diagnosed conditions (enthesopathy or sesamoiditis) were anatomically distant from the midfoot region assessed in this study. Ethical approval for this study was obtained from the Institutional Review Board of Taichung Veterans General Hospital (CE201886B). Demographic and clinical characteristics (age, gender, body weight, height, and BMI), as well as image data, were retrieved from the patients’ medical records and the gamma camera workstation. The flowchart of this study was demonstrated as Figure 1.

### 2.2. Radiographic Staging and Groups

**Figure 2:**
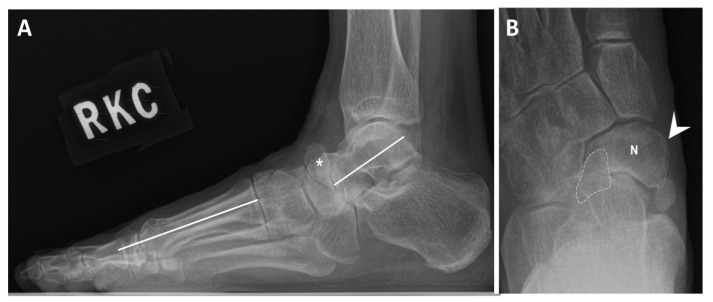
The measurement of Meary–Tomeno (M–T) angle at the lateral standing foot radiograph (solid white lines) (**A**) and radiographic features on DP view (**B**) of MWS foot in 49-year-old female. The measured M–T angle was 10.1 degree. This case was classified as Maceira stage 4 (asterisk: splitting of navicular; arrowhead: medial protrusion of navicular; N: navicular; dotted line: fragmentation of lateral navicular).

Maceira classification of Mueller–Weiss syndrome based on the changes in bone alignment on the lateral view of standing X-ray was used in the study [2]. Maceira classification represents structural severity based on radiographs, ranging from subtle changes (stages 1–2) to fragmentation/collapse (stages 3–4) and complete extrusion (stage 5). Foot deformity was measured according to the orientation of the Meary–Tomeno angle (M–T angle), i.e., the angle between the intersection of the talar and first metatarsal axes (Figure 2). Meary–Tomeno angle is considered normal within the range of −4° to 4° [20]. Five stages of MWS were reported by Maceria in 2004. Briefly, stage 1 exhibited minimal or no changes on radiographs. MRI may show intra-osseous edema. Stage 2 exhibited dorsal angulation of M–T line. The talar head appears dorsally subluxed. Stage 3 exhibited compression or splitting of navicular; M–T alignment is neutral. Stage 4 exhibited compression or splitting of navicular, as well as plantarwards M–T lines. Stage 5 exhibited the formation of talocuneiform articulation, complete extrusion of navicular and structural collapse.

The patient cohort was divided into control group and MWS group, according to the presence of disease. MWS group was further categorized into three subgroups based on Maceira classification, including mild grade: stage 1 and 2; moderate grade: stage 3 and 4; and severe grade: stage 5 groups, based on Maceira staging of lateral radiographs.

### 2.3. Images Acquisition

The Tc-99m MDP SPECT/CT scans were conducted using a Discovery NM/CT 670 Pro scanner (GE Healthcare, Milwaukee, WI, USA). Two hours after the injection of 740 MBq (20 mCi) of Tc-99m MDP, static plantar foot scintigraphy was obtained utilizing a large-field-of-view camera with a 256 × 256 matrix, a zoom factor of 1.46, and a 20% window centered on the 140 keV photopeak. A high-resolution collimator was employed (with a system resolution of 7.8 mm full width at half-maximum at a 10 cm distance; 165 cpm/μCi).

The SPECT images were acquired in a step-and-shoot mode, comprising 120 projections with 60 per detector head. Each projection lasted 12 s and was captured using a 128 × 128 matrix. The procedure was performed in room 1 with a 3-degree step. SPECT/CT data were reconstructed using the GE Q.Metrix algorithm, which incorporates attenuation correction, scatter correction, and resolution recovery, consistent with quantitative SPECT protocols. The SPECT data were reconstructed with CT-based attenuation correction derived from the low-dose CT component, scatter correction using an energy window-based method, and resolution recovery with modeling of the collimator–detector response. These corrections were applied routinely to all datasets before SUV calculation. No contrast medium was administered during the procedure. The SPECT/CT data were analyzed on the workstation, yielding transaxial, sagittal, and coronal slices of SPECT, CT, and fused SPECT/CT images. The interpretation of the Tc-99m MDP planar and SPECT/CT images was conducted by consensus between two experienced nuclear medicine physicians. Consensus readings by two nuclear medicine physicians were used to reduce inter-observer variability. Their evaluations were performed in a blinded manner, without information of the clinical grouping or Maceira stage when defining VOIs and assessing uptake intensity.

**Figure 3:**
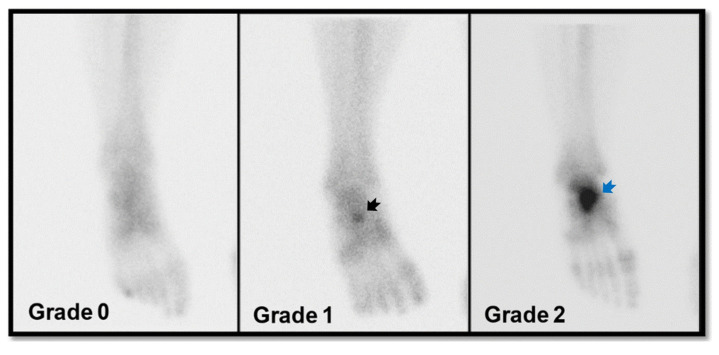
Visual grading criteria for Tc-99m MDP uptake in the navicular bone on conventional planar image of Tc-99m MDP bone scintigraphy. Grade 0: no visible focal uptake in the navicular region; Grade 1: mildly increased uptake compared with adjacent tarsal bones (black arrow); Grade 2: markedly increased focal uptake clearly exceeding the intensity of surrounding osseous structures (blue arrow).

The visual intensity (Vi) of tracer uptake of Tc-99m MDP bone scintigraphy in the navicular bones (N Vi), talus–calcaneus junction (TCj Vi), and calcaneus–cuboid joint (CCj Vi) were categorized into three groups as follows: Grade 0, indicating no discernible tracer uptake; Grade 1, denoting mildly increased tracer uptake; and Grade 2, signifying significantly increased tracer uptake (Figure 3).

**Figure 4:**
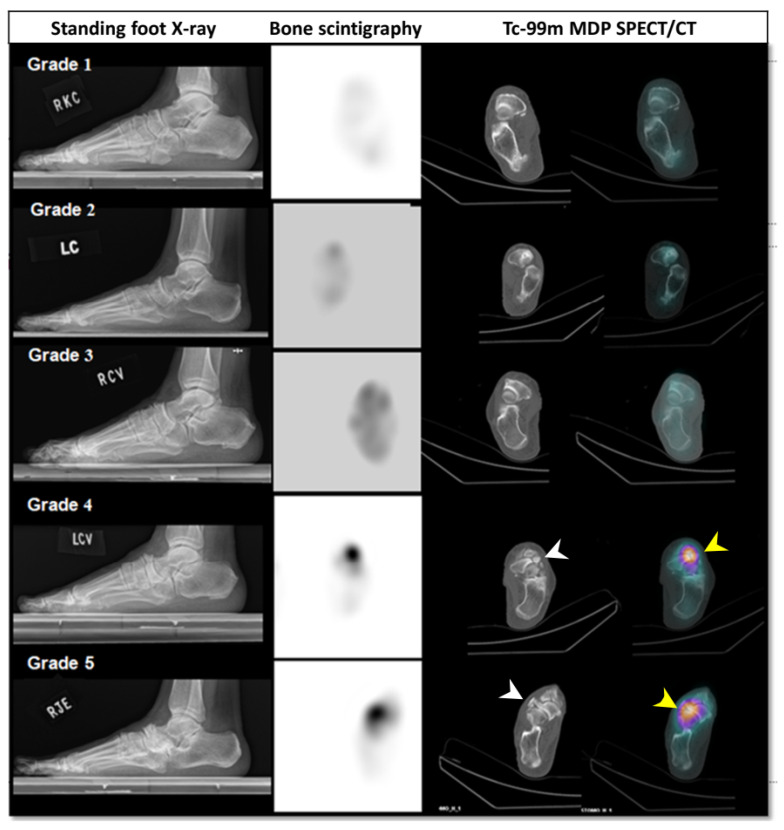
The associated changes in the images of bone scintigraphy and SPECT/CT in MWS cases of different Maceira stages. The white arrowheads in SPECT/CT panels indicate the navicular fragmentation and deformity. The significant higher radiotracer uptake at talonavicular joint in SPECT/CT panels (yellow arrowheads).

The associated changes on images of bone scintigraphy and SPECT/CT in MWS cases classified as Maceira stage 1–5 based on the weight-bearing lateral radiographs were demonstrated in Figure 4.

### 2.4. Quantitative Method of SPECT

**Figure 5:**
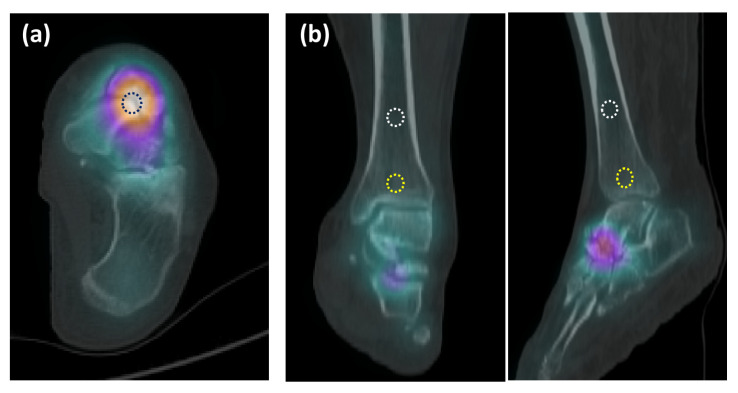
VOI was delineated on SPECT/CT images in MWS cases. (**a**) VOI on the lateral aspect of navicular bone presented as a dashed black circle; (**b**) a dashed yellow circle and the white circle signifies the VOIs on the tibial metaphysis (Tm) and tibial diaphysis (Td), positioned at the distal tibia, respectively. The color changes at TN joint in SPECT/CT indicate high radiotracer uptake.

For the quantitative analysis of Tc-99m MDP uptake, we employed the GE Q. Matrix reconstruction algorithm. Volume of interest (VOI) was delineated over the lateral aspect of navicular bone, as depicted in Figure 5a. The software automatically determined the maximum SUV (SUV_max_) within each VOI placed over the navicular bones. The standardized uptake value (SUV), a semi-quantitative measure of radiotracer uptake, was calculated using the following formula:

$$SUV=\frac{tissue\;radioactivity\;concentration\;[nCi/mL]}{\left[injected\;dose\;(mCi)/patient\;weight\;(g)\right]}$$

$$N/Tm\;ratio=\frac{navicular\;SUVmax}{tibial\;metaphysis\;SUVmax}$$

$$N/Td\;ratio=\frac{navicular\;SUVmax}{tibial\;diaphysis\;SUVmax}$$

To minimize subjective bias, we established specific guidelines for setting VOIs in the images. Initially, the VOI for MWS lesion of the navicular (N) was positioned in the lateral part of the navicular to avoid any interference from nearby joints and adjacent tarsal bones. The maximum value of SUV (SUV_max_) was measured as the highest pixel SUV within the designated VOI. VOIs for the tibial metaphysis (Tm), located 1 cm above the ankle joint surface, and the tibial diaphysis (Td), positioned at the midshaft of tibia, were also created (Figure 5b). SUV_max_ values were obtained for each VOI and used in data analysis. In cases where there was no apparent localized increase in radioactivity, the VOI was placed to correspond with the regions on the CT scan, and the maximum value was measured accordingly. In certain cases, abnormally increased MDP uptake was observed at the calcaneus–cuboid joint (CCj) or the talus–calcaneus junction (TCj). VOIs for these certain regions were also delineated and SUV_max_ values were likewise measured.

Furthermore, to maintain consistency, we set the average volume of VOIs for the navicular at 0.5 mL and for the tibia at 1 mL, according to predefined and standardized criteria. All procedures were carried out by an experienced technician using the GE Xceleris 4.0 workstation. The uptake ratios between the VOIs were then calculated and expressed as “N SUV_max_/tibial SUV_max_” (N/Tm ratio or N/Td ratio). This quantitative methodology closely aligns with and is based on a previous study [21].

### 2.5. Statistical Analysis

The variables analyzed in this study encompassed age, sex, body weight, body height, BMI, and SPECT/CT parameters of the feet. Data analysis was carried out using SPSS 22.0. Measurement data were presented as mean values (range). To assess the relationship between WMS cases and the control group, Mann–Whitney U tests were performed. Kruskal–Wallis tests were employed to evaluate the relationship between SPECT/CT parameters and the different groups of Maceira grades.

For the assessment of the predictive performance of SPECT/CT parameters, we utilized the receiver operating characteristic (ROC) curve methodology for censored SPECT/CT values, with the area under the ROC curve (AUC) serving as the criteria. We employed the SUV_max_-ROC method and the N/Tm, N/Td ROC method to evaluate the sensitivity, specificity, and accuracy of diagnosing Mueller–Weiss syndrome. Various cutoff values for SUV_max_ were determined through ROC analysis. All statistical tests were two-sided, and *p*-values less than 0.05 were considered statistically significant.

## 3. Results

### 3.1. Clinical Characteristics

A total of twenty-five participants, comprising 30 feet in 21 MWS cases and 10 feet of 5 controls (20.0%), were included for analysis in the study. Table 1 presents the characteristics of all subjects and the correlation between WMS cases and the control group. For each of the variables (age, gender, body height, body weight, and BMI), there are no significant differences between subgroups for these variables (Table 1).

### 3.2. Radiographic and SPECT/CT Measures in Subgroups

The M–T angle measured on the lateral view of foot X-ray has a median of 12.3 degrees, ranging from 4.6 to 16.5 degrees. The *p*-value (*p* < 0.001) indicates a highly significant difference in M–T angle between the control and MWS groups. Visual analysis on planar images of bone scintigraphy also showed significant difference in N Vi between the two groups (<0.001).

Among SPECT/CT parameters, the control group has a median N SUV_max_ of 1.5, ranging from 1.2 to 2.3, while the MWS group has a median N SUV_max_ of 9.2, ranging from 4.6 to 27.7. The *p*-value is highly significant (<0.001), showing a significant difference in N SUV_max_ between the control and case groups.

### 3.3. Subdivided MWS Group Based on Maceira Grades

The MWS group was further subdivided into three subgroups based on Maceira grades (Stage 1/2, Stage 3/4, and Stage 5). The same variables of SPECT/CT are analyzed within these subgroups. The N Vi, N SUV_max_, N/Tm ratio, and N/Td ratio of the Stage 5 group were the highest with statistical significance (*p* < 0.001) among those of the control group and the Maceira Stage 1–2, 3–4 groups. Certain variables, such as N Vi, TCj VI, CCj VI, N SUV_max_, Tm SUV_max_, and N/Tm Ratio, have significant positive correlations with both M–T angle and Maceira grade. In our cohort, these structural and metabolic parameters show a stepwise correlation. Early Maceira stages (1–2) typically demonstrate Grade 1 uptake on planar images with moderately increased SUV_max_ values. Intermediate stages (3–4) more often show Grade 1–2 uptake with markedly higher SUV_max_, consistent with increased bone turnover in progressively deformed bone. In the most advanced stage (5), planar imaging consistently shows Grade 2 uptake with the highest SUV_max_ values, reflecting intense remodeling in severely collapsed or extruded navicular bone. Additionally, it is worth noting that age and BMI do not exhibit statistically significant correlations with M–T angle or Maceira grade in this dataset (Table 2).

### 3.4. Diagnostic Performance of Measures on SPECT/CT

**Figure 6:**
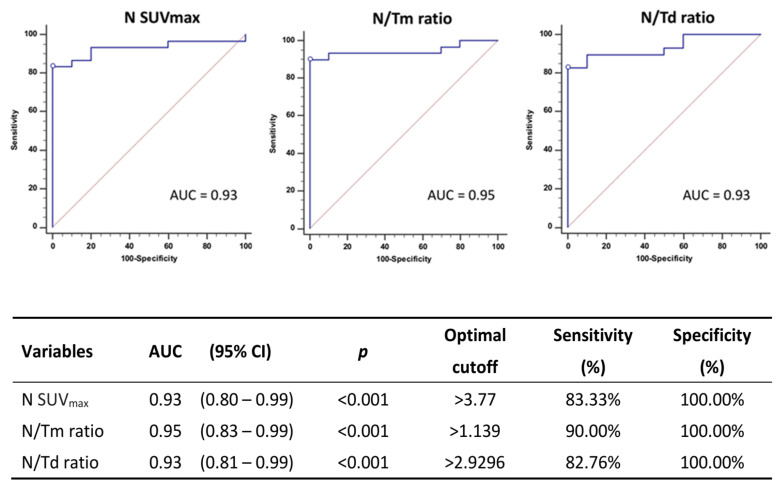
ROC curve and diagnostic performance of three different variables (N SUV_max_, N/Tm ratio, and N/Td ratio) measured from SPECT/CT.

ROC curves for SPECT/CT parameters in diagnosing WMS are shown in Figure 6. The ROC curves were generated using the values of N SUV_max_, N/Tm ratio, and N/Td ratio to determine their optimal cutoff values. Patients with a N SUV_max_ greater than 3.77 (sensitivity of 83.33%, specificity of 100.0%, and AUC 0.93), N/Tm ratio greater than 1.139 (sensitivity of 90.00%, specificity of 100.0%, and AUC 0.95), or an N/Td ratio greater than 0.93 (sensitivity of 82.76%, specificity of 100.0%, and AUC 0.93), respectively, demonstrated a diagnosis of WMS. The N SUV_max_, N/Tm ratio, and N/Td ratio are effective diagnostic variables for MWS with high AUC values and excellent sensitivity and specificity. The optimal cutoff values help clinicians easily determine whether a patient is likely to have MWS based on these variables.

### 3.5. Correlations Between Parameters and M–T Angle and Maceira Stage

The parameters including age, BMI, visual intensity of VOIs on bone scintigraphy, and quantitative and semi-quantitative values on SPECT/CT were evaluated in correlation to M–T angle and Maceira stage. Neither age nor BMI demonstrated a significant correlation with disease severity, while metabolic SPECT/CT parameters strongly reflect disease severity across Maceira stages. The N Vi, TCj Vi, CCj Vi, N SUV_max_, and N/Tm ratio were positively correlated to the M–T angle, but not Tm SUV_max_ and Td SUV_max_. However, only the factors, N Vi, N SUV_max_, N/Tm ratio, and N/Td ratio were positively correlated to MWS severity based on Maceira stage. (Table 3)

## 4. Discussion

Muller–Weiss syndrome, being a rare disease, has limited large-scale studies regarding disease diagnosis and treatment. Further discussion of MWS is warranted due to the scarcity of information. In this study, we have enrolled relatively more MWS cases (thirty MWS feet) compared to the previous research and first attempted to elucidate the feasibility of quantification or semi-quantification of the radiotracer uptake on bone scintigraphy with SPECT/CT, comprising N SUV_max_, N/Tm ratio, and N/Td ratio, for MWS diagnosis. The optimal cutoff values of N SUV_max_, N/Tm ratio, and N/Td ratio with very high sensitivity and specificity in ROC curve for MWS were 3.77, 1.139, and 0.93, respectively. These findings indicated the measures on bone scintigraphy with SPECT/CT, either quantified or semi-quantified, help the clinicians look for deeper insights for MWS.

As stated in previous reports, higher BMI, obesity, or middle-aged women might be the susceptible risk factors for MWS [22], as reflected in our study with an average age of 65 and more females (70.0%) in MWS cohort, consistent with similar findings in other studies [23]. However, weight and BMI do not show a correlation with MWS in the current study; it is inconsistent with the other study and may necessitate much more cases to determine whether disease prevalence is unrelated to obesity or chronic repetitive damage [24]. Although MWS often affects both feet, the disease can manifest at different stages with non-simultaneous symptoms [25]. In our cohort, six feet were classified as Maceira stage I, without significant bony disintegration on radiographic findings of X-ray and CT scan. The diagnosis for early-stage MWS is challenging and easily misdiagnosed through radiographs but it can be effectively identified through SPECT/CT. Furthermore, MWS may not occur simultaneously and the severity may vary. These six patients in this current study have mild grade (stage 1/2) in one foot and moderate grade (stage 3/4) in the other. SPECT/CT scans can easily identify the early-stage MWS by significantly enhanced lesion localization. The detection of early stage of MWS may help clinicians to offer treatment to prevent disease progression. Ruiz-Escobar et al. reported insole support was effective for all stage 2 of MWS to avoid surgical treatment in their case series [26]. However, the impact of subsequent treatment strategies for early MWS diagnosis needs to be further investigated on a larger scale.

SPECT/CT imaging helps identify lesions by showing abnormal Tc-99m MDP accumulation at the lateral part of the navicular bone or adjacent joints, such as the naviculocuneiform joints, talocalcaneal junction, or calcaneocuboid joint. This information is crucial for disease diagnosis and for tailoring the surgical arthrodesis site [25,27]. Considering that the susceptible population may consist of middle-aged individuals, potential associations with obesity, diabetes, or menopausal changes should be evaluated. These factors need to be differentiated from other prevalent conditions that cause foot arch pain, such as talus necrosis or Charcot foot. Abnormal Tc-99m MDP accumulation in SPECT/CT images not only signifies bone destruction but also correlates with increased blood flow, congestion, or local inflammation. It also aligns with clinical pain locations [28]. Therefore, SPECT/CT imaging holds clinical value for confirming the diagnoses, excluding other causes, and providing effective treatment.

Various hypotheses have been proposed to explain the pathogenesis of MWS; however, its underlying mechanisms remain incompletely understood. In our cohort, visual analysis of SPECT/CT images enabled early detection of abnormal MDP uptake, and the visual intensity grades, SUVs, and SUV ratios demonstrated significant correlations with clinical severity. In more advanced stages of MWS, the marked bony destruction and deformity seen on radiographs and CT were accompanied by pronounced Tc-99m MDP accumulation, suggesting substantially increased bone turnover in the affected region. Notably, even in early-stage MWS with minimal radiographic abnormalities, localized increases in MDP uptake at the lateral navicular were readily identified on visual, quantitative, and semi-quantitative assessments. These findings imply that metabolic alterations may precede overt structural changes in the disease course.

Notably, the pattern of increased radiotracer uptake observed in MWS differed from that typically seen in classical avascular necrosis (AVN), e.g., AVN of the femoral head. In AVN, SPECT commonly shows photopenic areas corresponding to ischemia, with reactive uptake detected inferior to the necrotic zone [29,30]. The recent literature has also highlighted how certain osteochondral conditions previously labeled as spontaneous osteonecrosis, such as “spontaneous osteonecrosis of the knee (SONK)”, are now recognized as subchondral insufficiency fractures [31]. Drawing from this evolving understanding, our findings suggest that MWS may likewise involve pathological mechanisms other than primary avascular necrosis. Factors such as mechanical overload, repetitive microtrauma, congenital dysplasia, or indolent chronic inflammation may contribute to the disease process. While a vascular component cannot be entirely excluded, the imaging and metabolic characteristics observed in our study do not fully align with a purely ischemic AVN paradigm. Further studies will be necessary to clarify these mechanisms.

In this current study, three different kinds of measures were first employed for SPECT image analysis of MWS lesions: visual analysis with visual intensity, quantitative values, N SUV_max_, and semi-quantitative values including N/Tm ratio and N/Td ratio. All three approaches have statistical significance and can aid in diagnosis using appropriate cut-off values. According to our results, the performance of AUC in assisting the diagnosis of MWD using semi-quantitative values such as N/Tm ratio or N/Td ratio is slightly superior to that of N SUV_max_. As we previously discovered in earlier studies, semi-quantitative values in nuclear medicine imaging exhibit high correlation with clinical symptoms in lupus nephritis, serving its potential as a non-invasive and effective assessment tool [21]. In comparison to measuring absolute quantitative values on SPECT images, obtaining retrospective semi-quantitative values allows for easier execution, reducing the difficulties, time, and manpower involved during the examination process [32]. Furthermore, relying on the radiotracer uptake on patient’s own distal tibia as a reference for semi-quantitative values tends to minimize individual differences, mitigating interference from factors such as radionuclide dosage or diverse metabolism of the patients. According to our results, there are no significant differences on Tm SUVs and Td SUVs between normal population or in patients with different severity levels of MWS. This implies that the MDP uptake in the tibia is relatively stable and can serve as a reliable reference of baseline. Semi-quantitative values, N/Tm ratio, or N/Td ratio are easier and more convenient measures in clinical practice compared to quantitative values and demonstrate good diagnostic capabilities for disease discrimination of MWS. These two semi-quantitative measures supply alternative methods to evaluate the MWS; however, whether they can completely replace quantitative values requires further investigation.

In clinical practice, the SUV_max_ and ratio-based thresholds identified in this study may help differentiate MWS from other causes of midfoot pain, especially when radiographic findings are subtle. However, SUV measurement may vary across different scanners, reconstruction protocols, and institutions. Although these SPECT/CT thresholds showed good diagnostic performance, they may not apply universally across all clinical settings. SPECT/CT metrics should be interpreted within a broader clinical context, including symptom severity, functional status, comorbidities, and patient preference rather than be used as standalone guides for treatment decisions.

There are some limitations in this study. First, due to the retrospective study design, the selection bias or unknown confounding factors may not be excluded. Second, our study is limited by the relatively small sample size due to the rarity of Muller–Weiss syndrome, especially the Stage 5 subgroup. Further research with larger database might be required to verify our results. Larger, multi-center datasets would be beneficial for validating the proposed SUV_max_ and ratio cutoffs and improving generalizability given the rarity of MWS. Lastly, no functional outcomes were available in this study and there were no correlations between the SPECT/CT variables and functional outcomes. Further studies should be conducted for their clinical significance.

## 5. Conclusions

The direct measure of SUVs on lateral navicular bone or semi-quantitative parameters, N/Tm ratio, and N/Td ratio from SPECT/CT images can be valuable in the diagnosis of Mueller–Weiss syndrome with great diagnosing accuracy. Especially, the SUV or ratio measures through SPECT/CT help to detect the subtle or nonexistent changes in radiographs in the early stage of MWS. This could provide clinicians an effective guidance for timely management and further treatment strategies. The utilization of Tc-99m MDP SPECT/CT scans is highly recommended and offers further insights into MWS.

## Figures and Tables

**Table 1:** The characteristics of cases between control and MWS groups.

	Control (*n* = 5)	MWS (*n* = 21)	*p* Value
Age	57.0 (29.5–71.0)	65.0 (59.0–68.5)	0.312
Gender			0.302
Female	2 (40.0%)	15 (71.4%)	
Male	3 (60.0%)	6 (28.6%)	
Body height	159.0 (148.5–167.0)	157.0 (152.0–161.0)	0.974
Body weight	57.0 (54.0–85.0)	65.0 (57.0–73.5)	0.313
BMI	22.8 (21.9–32.6)	27.4 (23.2–29.1)	0.558

Chi-square test or Mann–Whitney U test. Median (IQR).

**Table 2:** Comparisons of SUV parameters and visual intensity between feet in control and MWS groups.

	Control (*n* = 10)		MWS (*n* = 30)		*p* Value
Maceira stage	--		4.0 (1.0–4.0)		
M–T angle	−0.7 (−10.3–3.1)		12.3 (4.6–16.5)		<0.001 **
N Vi	0.0 (0.0–0.0)		1.5 (1.0–2.0)		<0.001 **
TCj Vi	0.0 (0.0–0.0)		0.0 (0.0–1.0)		0.335
CCj Vi	0.0 (0.0–0.0)		0.0 (0.0–1.0)		0.016 *
N SUV_max_	1.5 (1.2–2.3)		9.2 (4.6–27.7)		<0.001 **
Tm SUV_max_	2.1 (1.9–2.7)		2.9 (2.0–3.8)		0.118
Td SUV_max_	1.3 (0.8–1.8)		1.4 (0.9–1.8)		0.662
N/Tm ratio	0.8 (0.6–0.9)		4.8 (1.7–8.7)		<0.001 **
N/Td ratio	1.5 (0.9–1.9)		10.3 (3.7–17.8)		<0.001 **
	**Control** (*n* = 10 feet)	**Stage 1/2** (*n* = 10 feet)	**Stage 3/4** (*n* = 16 feet)	**Stage 5** (*n* = 4 feet)	***p* Value**
N Vi	0.0 (0.0–0.0)	1.0 (0.8–1.3)	2.0 (1.0–2.0)	2.0 (2.0–2.0)	<0.001 **
TCj Vi	0.0 (0.0–0.0)	0.0 (0.0–0.0)	0.0 (0.0–0.0)	1.0 (1.0–1.0)	0.005 **
CCj Vi	0.0 (0.0–0.0)	0.0 (0.0–0.3)	0.0 (0.0–1.0)	1.0 (1.0–1.8)	0.002 **
N SUV_max_	1.5 (1.2–2.3)	5.6 (3.1–10.2)	18.0 (4.7–39.3)	22.5 (21.2–23.9)	<0.001 **
Tm SUV_max_	2.1 (1.9–2.7)	2.9 (1.6–3.7)	2.8 (2.1–4.3)	3.5 (2.3–4.4)	0.365
Td SUV_max_	1.3 (0.8–1.8)	1.4 (0.9–2.3)	1.5 (1.2–1.8)	0.9 (0.6–1.4)	0.270
N/Tm ratio	0.8 (0.6–0.9)	1.7 (1.0–5.2)	6.3 (1.9–9.5)	6.7 (5.0–10.3)	<0.001 **
N/Td ratio	1.5 (0.9–1.9)	3.4 (1.9–7.9)	15.2 (4.1–18.8)	25.2 (16.5–36.8)	<0.001 **

Chi-square test or Mann–Whitney U test. Median (IQR) * *p* < 0.05, ** *p* < 0.01. M–T angle = Meary–Tomeno angle; N = navicular; Vi = visual intensity; TCj = talus–calcaneus junction; CCj = calcaneus–cuboid joint; Tm = tibial metaphysis; Td = tibial diaphysis.

**Table 3:** The parameters correlate to M–T angle and Maceira stage.

	M–T Angle	Maceira Stage
	r_s_	r_s_
Age	0.091	0.099
BMI	0.015	−0.007
N Vi	0.618 **	0.544 **
TCj Vi	0.462 *	0.231
CCj Vi	0.506 **	0.258
N SUV_max_	0.491 **	0.438 **
Tm SUV_max_	0.190	0.202
N/Tm ratio	0.537 **	0.439 **
Td SUV_max_	−0.183	−0.003
N/Td ratio	0.671 **	0.485 **

Spearman’s rho coefficient. * *p* < 0.05, ** *p* < 0.01. M–T angle = Meary–Tomeno angle.

## Data Availability

The original data presented in the study are openly available in FigShare at DOI 10.6084/m9.figshare.30572747.

## Abbreviations

The following abbreviations are used in this manuscript:

MWS	Mueller–Weiss Syndrome
Tc-99m MDP	Tc-99m-labeled methylene diphosphonate
SPECT	Single photon emission computed tomography
CT	Computed tomography
SUV	Standardized uptake value
BMI	Body Mass Index
M-T angle	Meary–Tomeno angle
Vi	Visual intensity
N Vi	Visual intensity in navicular bones
TCj Vi	Visual intensity in talus–calcaneus junction
CCj Vi	Visual intensity in calcaneus–cuboid joint
VOI	Volume of interest
Tm	Tibial metaphysis
Td	tibial diaphysis
ROC	receiver operating characteristic
